# Improving Health Outcomes in Coronary Artery Disease Patients with Short-Term Protocols of High-Intensity Interval Training and Moderate-Intensity Continuous Training: A Community-Based Randomized Controlled Trial

**DOI:** 10.1155/2023/6297302

**Published:** 2023-12-18

**Authors:** Catarina Gonçalves, Jorge Bravo, João Pais, Ana Abreu, Armando Raimundo

**Affiliations:** ^1^Department of Sports and Health, School of Science and Technology, University of Évora, 7000-727 Évora, Portugal; ^2^Comprehensive Health Research Centre (CHRC), Portugal; ^3^Department of Cardiology, Espírito Santo Hospital of Évora, 7000-811 Évora, Portugal; ^4^Department of Cardiology, Santa Maria Hospital, 1649-028 Lisbon, Portugal

## Abstract

Studies have shown that the higher the aerobic capacity, the lower the risk of cardiovascular mortality and morbidity. In the case of cardiac patients, high-intensity interval training (HIIT) seems to be more effective than moderate-intensity continuous training (MICT) in improving aerobic capacity. The aim of this study was to investigate the effects of two community-based exercise programs using two short-term protocols (HIIT and MICT) on physical fitness and physical activity (PA) levels in coronary artery disease (CAD) patients. *Methods.* In this randomized controlled trial, body composition, aerobic capacity, muscle strength, and daily PA levels were assessed before and after 6 weeks of intervention in 69 patients diagnosed with CAD. All patients were randomly (1 : 1 : 1) assigned to two exercise groups (HIIT or MICT) or a control group (no exercise). Both training programs consisted of 6 weeks of supervised treadmill exercise, three sessions per week. MICT targeted ≈70-75% of peak heart rate (HR), while HIIT aimed for ≈85-95% of peak HR. The control group only followed the medical recommendations. *Results.* Community-based exercise programs showed more positive effects on physical fitness variables and physical activity levels compared to control. HIIT could significantly improve waist circumference, body fat mass, VO_2_peak, sedentary behavior, and moderate-to-vigorous PA compared to MICT. Moreover, the control group showed poorer results. *Conclusion.* HIIT can improve health outcomes more positively than MICT and control. These findings indicate that HIIT may be an alternative and effective training method in community-based exercise programs for CAD patients. This trial is registered with NCT03538119.

## 1. Introduction

Cardiovascular disease (CVD) stands as the predominant global cause of mortality, contributing to a substantial 30% of all recorded deaths (16.7 million individuals) [1]. In Portugal, cardiovascular disease accounts for 29.5% of all deaths, highlighting its importance in public health and the need for preventative measures [2]. Cardiac rehabilitation (CR) has garnered worldwide acceptance as a potent secondary prevention tool in secondary prevention of CVD. CR programs are aimed at increasing the aerobic capacity and muscular strength of patients with CVD [3]. Aerobic capacity is recognized as a robust indicator of cardiovascular health and a well-established predictor of total and cardiovascular mortality in people with and without CVD. As a reference, improving aerobic capacity by 3.5 mL kg^−1^ min^−1^ is associated with a ∼15% reduction in coronary heart disease/cardiovascular-related mortality [4]. Fallavollita et al. [5] studied coronary artery disease (CAD) patients who participated in a 5-week CR program and found that CR improved aerobic capacity. Similarly, Kim et al. [6] observed that a 6-week CR exercise program, with an intensity of 60-85% heart rate reserve, also improved aerobic capacity. In addition, resistance training has been found to enhance muscle strength and endurance and positively impact cardiovascular risk factors, metabolism, and cardiovascular function in patients with heart disease [7–10]. Previous studies have shown that exercise-based CR is also beneficial for improving blood pressure [2, 11], blood lipids [2, 11], physical fitness [12, 13], body composition [14–16], and health-related quality of life [5, 17, 18].

Moderate-intensity continuous training (MICT) has traditionally been considered a fundamental part of aerobic-based exercise prescription, performed at an intensity between 50 and 75% of the heart rate (HR) [19]. This type of training has proven to be effective in improving the cardiovascular health of patients with CVD both in the short and the long term [20]. However, high-intensity interval training (HIIT) has recently emerged as an alternative or complementary strategy to MICT. HIIT involves repeated bouts of relatively higher-intensity exercise (between 85 and 100%) intermingled with intervals of lower-intensity recovery [21]. Studies have shown that HIIT can lead to similar or even greater improvements in VO_2_peak when compared to MICT [20, 22, 23]. Precisely, multiple recent meta-analyses [20, 24, 25] exploring the efficacy of HIIT within CVD patients have reported more remarkable improvement in aerobic capacity compared to MICT. Some studies have shown strong evidence that HIIT is an effective method for improving strength [26, 27], gait [26, 28], and body composition [16, 29] in CAD patients. In fact, there is a trend indicating that HIIT may have more beneficial effects on health indices and markers than MICT [30].

Patients with CAD are encouraged to uphold an active lifestyle after the CR. Nevertheless, during the observation stage after the completion of exercise-based CR, the patient's adherence to exercise remains low [31], and physical activity (PA) engagement decreases drastically [32, 33]. It is recommended by major healthcare organizations that CR patients accumulate 30 to 60 minutes of moderate-intensity PA per day on more than 5 days of the week and reduce the amount of time spent in sedentary behavior (SB) [34].

The objective of this randomized controlled trial (RCT) was to compare the effectiveness of 6-week supervised community-based exercise protocols, a short-duration resting HIIT and a usual MICT in improving health indicators among CAD patients, specifically on physical fitness and physical activity levels.

## 2. Methods

### 2.1. Participant Selection and Allocation

**Figure 1:**
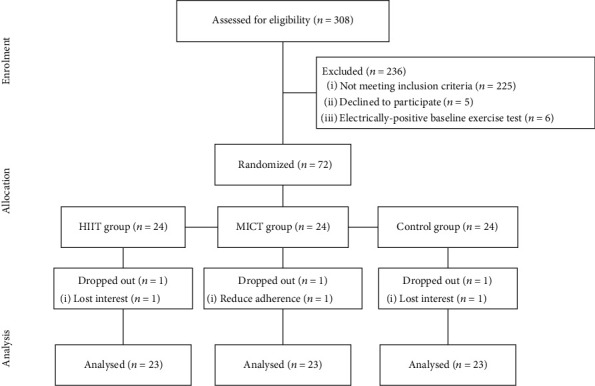
Diagram of the study.

Seventy-two patients (men and women) were recruited between March 2018 and November 2021 within the cardiology unit of the Espírito Santo Hospital of Evora (Portugal). The study included patients who had suffered a coronary event and were referred to the community-based exercise programs by their cardiologist, two months after angioplasty. Patients between the ages of 18 and 80, with a left ventricular ejection fraction ≥45%, and classified as New York Heart Association (NYHA) functional class I or II were considered for inclusion. Patients who had severe exercise intolerance, uncontrolled angina pectoris, uncontrolled arrhythmia, lung or severe kidney diseases, musculoskeletal or neuromuscular conditions preventing exercise testing and training, and signs or symptoms of ischemia were excluded from the study. Patients underwent a medically supervised cardiopulmonary exercise test (CPET) baseline testing performed on a treadmill with the Bruce protocol, before 1 : 1 : 1 randomization to either HIIT or MICT or control (no community-based exercise program). The test was conducted in nonfasting conditions and under medication. During the test, an electrocardiography was continuously recorded, and blood pressure was measured every 3 minutes using an arm cuff. The functional capacity of the patient was determined in metabolic equivalent values (METs). Patients who showed abnormal results during the baseline CPET were excluded from the study, and further angiography was performed. Blood samples were drawn on the same day as CPET but were collected before exercise in fasting conditions. After completion of the informed consent process, blood sample, medication, and clinical history are obtained from all patients to evaluate the eligibility criteria (Figure 1).

After baseline testing, the patients were enrolled in the trial and given a trial-specific identification number (ID). The three groups were similar concerning age, coronary risk factors, type of coronary event or left ventricular ejection fraction, and extent of coronary artery disease. We chose to carry out a short-term (6-week) program based on the systems of other countries, particularly Australia, Hungary, and Austria [35].

### 2.2. Health Outcome Measures and Assessments

The patients were submitted to a clinical evaluation of physical fitness (body composition, aerobic capacity, and muscle strength) and physical activity (by accelerometry), performed by a physiologist at the University of Evora laboratory. Patients were asked to bring any medications that they were taking to the assessments. Initially, each patient completed a standardized questionnaire including demographic data, family history of CVD, smoking status medication use, and medical history.

#### 2.2.1. Physical Fitness (Body Composition, Aerobic Capacity, and Muscle Strength)

Body mass index (BMI) was calculated directly by the standard formula: weight (kg)/height (m)^2^. The waist circumference was manually measured according to the standard procedures of ACSM guidelines [36, 37]. Body composition was then evaluated using dual-energy X-ray absorptiometry (DXA) scans, performed with QDR 2000 densitometers (Hologic QDR, Hologic, Inc., Bedford, MA, USA) in array beam mode. The scans took place one week prior to and following the completion of 18 exercise sessions. These scans were used to determine total body mass, body fat mass, body lean mass, body fat percentage, and abdominal region fat percentage (defined as the area between the ribs and the pelvis by GE HealthCare systems). Daily calibration of the scanner was completed using a manufacturer-supplied calibration block to ensure accuracy and control for potential baseline drift.

Aerobic capacity was characterized as peak oxygen consumed (VO_2_peak, ml·kg^−1^·min^−1^). The VO_2_peak was calculated from the equation VO_2_peak = 4.9486 + 0.023 × walk distance (meters) that was determined via using the six-minute walking test (6MWT) [38]. The 6MWT was performed in a 50 m premarked University of Evora pavilion, and instructions and encouragement were given following the test's guidelines [39]. This test is well validated for CAD patients and has demonstrated good reliability in this group of patients [40].

An isokinetic dynamometer (Biodex®, System 3 Pro, Biodex Corp., Shirley, NY, USA) was used to measure isokinetic muscle strength, utilizing the “unilateral concentric protocol” for the dominant knee extensor and flexor muscles. Patients were tested in a sitting position with hip flexion and using stabilization straps applied to the trunk, waist, and thigh. Peak torque (three repetitions) and fatigue resistance (20 repetitions) were evaluated at angular velocities of 90°/s and 180°/s of the dominant knee. The peak torques of the knee extensor and flexor muscles were adjusted by body weight according to the formula strength (Nm) × 100/body weight (kg) since it is well known that the peak muscle power is closely associated with body weight [41].

#### 2.2.2. Physical Activity Levels

After completing all clinical evaluations, patients were instructed to wear a triaxial accelerometer (ActiGraph GT3X) on their hip, placed anterior to the right iliac crest, for seven consecutive days during waking and sleeping hours. The accelerometer was removed during activities such as bathing or swimming. The three-axis acceleration data was processed using ActiGraph software (ActiLife, version 6) in 15-second epochs (raw data recorded at 30 Hz) using the standard filter. The data was then integrated into a vector magnitude count by taking the square root of the sum of squared axes (vertical, anterior-posterior, and medial-lateral). Daily averages (min/day) of accelerometer-measured PA were calculated for each patient and classified into five activity levels (sedentary time 1.00–1.99 MET, light PA 2.00–3.49 MET, and all activity ≥ 3.50 MET classified as moderate-to-vigorous PA) using the limits set by the manufacturer [42]. A valid day was defined as ≥10 hours of wear time. All activity with intensity 1 MET (1 Met = 3.5 ml·kg^−1^·min^−1^) or higher was calculated on wear time. Patients with at least four valid days (3 weekdays and 1 weekend day) were included in the analyses (monitor wear time of ≥600 min/day) [43].

All measurements were taken at the beginning and completion of 18 sessions of community-based exercise programs. The protocols of pre- and postintervention were the same for each patient.

### 2.3. Exercise Training Protocols

**Figure 2:**
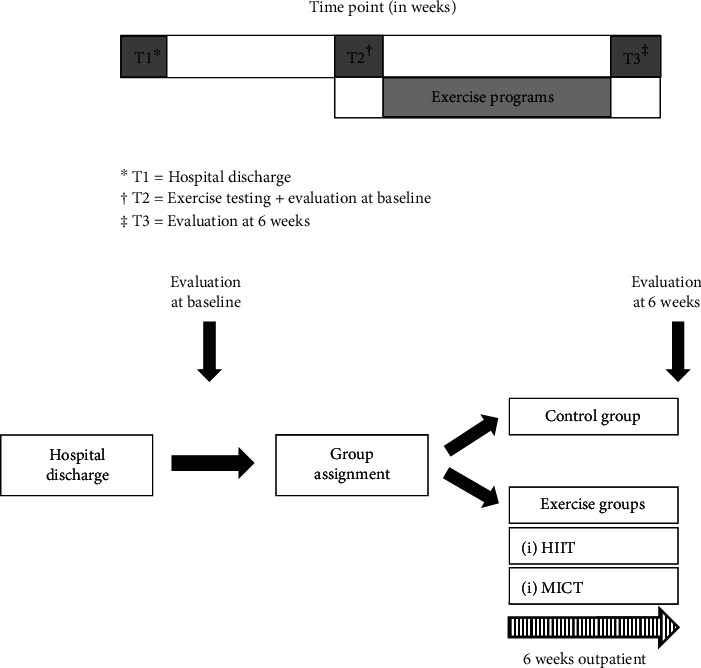
Study design and time frame. HIIT = high-intensity interval training; MICT = moderate-intensity continuous training; T = time point.

After hospital discharge, dietary advice, psychological support, and educational intervention were performed to all patients. The community-based exercise programs (HIIT and MICT) consisted of six weeks of supervised treadmill exercise, three sessions per week (Figure 2). If a session was missed, it was made up that week or the following week. Patients performed each exercise session alone or in a group, with a maximum of three patients per session. The control group did not receive any additional follow-up regarding exercise beyond general counseling on the importance of exercise and diet.

The 10-point category-ratio Borg scale [44], usually referred to as the rating of perceived exertion (RPE), was used to assess patients' perceived effort during exercise. The Borg scale is a 10-point scale ranging from 0 to 10 with anchors ranging from “no exertion at all” (0) to “maximal exertion” (10). Patients were asked to rate their exertion before (pre-exercise), minute to minute of exercise, and postexercise. Levinger et al. [45] and Buchheit and Laursen [46] demonstrated that the RPE has shown a great correlation with HR, ventilation, and VO_2_peak in individuals with and without CAD. Furthermore, this correlation is not affected by the use of beta-blockers, a common medication used to modulate heart rate in CAD patients [29].

**Figure 3:**
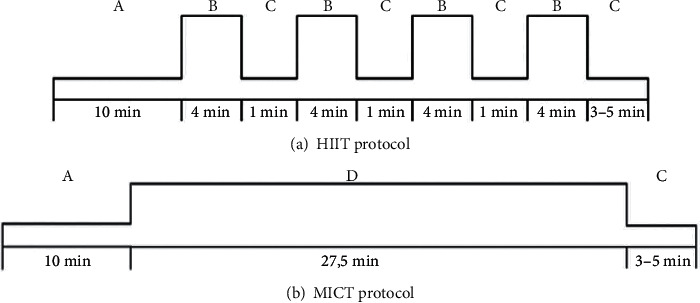
Summary of the exercise training protocol. HIIT = high-intensity interval training; MICT = moderate-intensity continuous training; min = minutes. A—Warm-up; B—interval bout of high intensity exercise; C—one-minute recovery interval; D—cooldown; E—continuous bout of moderate-intensity exercise.

Each exercise session was started with a 5–10-minute warm-up at 50–60% HRpeak and finished with 5 minutes of cooldown at 40% HRpeak. The HIIT group performed 4 × 4-minute high-intensity intervals at 85%–95% HRpeak followed by a 1-minute recovery interval at 40% HRpeak, predicted with the Bruce protocol [38, 47]. During the exercise, the patients were motivated to progressively increase their exercise intensity towards 6–9 (hard to very hard) on a 0 to 10 Borg scale [44]. The MICT group (traditional care) performed a continuous bout of moderate-intensity exercise at 70–75% HRpeak, rating perceived exertion 3 to 5 (fairly light to somewhat hard), for 28 minutes in order to equate the energy expenditure with the HIIT group (Figure 3).

The exercise intensity was calculated using the heart rate reserve equation (Target HR = [(HRmax − HRrest) × %intensity desired] + HRrest [36]), predicted with a supervised graded exercise test on a treadmill with the Bruce protocol [38]. HRR is defined as the difference between the basal rates of HR. Training sessions were supervised by a physiologist. As training intensity increased, the patient's heart rate, rate of perceived exertion (Borg's scale), and cardiac symptoms were also taken into consideration. Heart rates were observed with polar heart rate monitoring (Polar Electro Oy, Kempele, Finland), and blood pressure was measured at the beginning and the end of each session.

### 2.4. Ethical Considerations

This RCT followed the CONSORT guidelines for RCTs (http://www.consort-statement.org) and was conducted in accordance with the Declaration of Helsinki and registered at ClinicalTrials.gov (NCT03538119). The Ethics Committee of the University of Évora (reference number: 17039) has approved the study design, protocol, and informed consent procedure. All patients signed a written informed consent before participating in this study.

### 2.5. Statistical Analyses

The sample size was calculated using the online G^∗^Power software, considering an effect size of 0.3, a predefined sample power of 0.8, a predefined error probability defined as 0.05, and statistical power of 95%. Hence, a minimum sample size of 66 participants was determined (22 participants for each group) to identify significant changes. To test the normality and homogeneity assumptions, we used the Kolmogorov-Smirnov and Levene tests, respectively. As the majority of the sample variables did not follow a normal distribution, nonparametric statistical analyses were used. We conducted between-group comparisons using the Kruskal-Wallis test and within-group comparisons using the Friedman test; both tests were followed by post hoc pairwise comparisons (Bonferroni's correction). We calculated the means and standard deviations for all variables. To compare postintervention values with baseline values, we calculated the delta value (*Δ:* moment*_x_* – moment_*x-*1_) and the proportional change delta value (Δ% : [(moment_*x*_–moment_*x*−1_)/moment_*x*−1_] × 100) for all variables. The effect size (ES) was calculated using Cohen's method since the data were not normally distributed [48]. The ES was computed and classified based on Cohen's thresholds (small: *d* = 0.10; medium: *d* = 0.30; and large: *d* ≥ 0.50) [49]. Analyses were performed using the SPSS software package (version 24.0 for Windows, IMB Statistics). A value of *p* ≤ 0.05 was considered statistically significant for all analyses. A code was assigned to each patient to preserve their anonymity.

## 3. Results

Patient characteristics at baseline are described in Table 1. Baseline characteristics were not different for the HIIT, MICT, and control groups: age (50 ± 9 vs. 55 ± 10 vs. 57 ± 11 years, respectively), female (15% vs. 17% vs. 15%), BMI (28.2 ± 4.5 vs. 29.4 ± 3.9 vs. 29.4 ± 4.3 kg/m^2^), and waist circumference (98.4 ± 14.5 vs. 101.1 ± 10.3 vs. 101.1 ± 10.8 cm). Most patients were hypertensive and sedentary, with dyslipidemia and a family history of cardiovascular disease. The most common medications were statins, antiplatelet therapy, and *β*-blockers. Comorbidities and medications were also not different between groups (*p* > 0.05).

**Figure 4:**
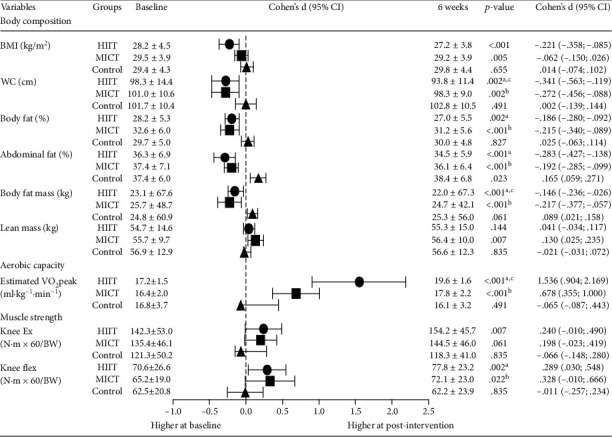
Physical fitness measurements of exercise groups (HIIT (*n* = 23) and MICT (*n* = 23)) and control group (*n* = 23). BMI = body mass index; Control = control group; Ext = extensors; Flex = flexors; HIIT = high-intensity interval training; MICT = moderate-intensity continuous training; WC = waist circumference. Values are reported as mean ± standard deviation; 95%CI = 95% confidence interval. ^a^Significant differences between HIIT and control, *p* < 0.05; ^b^significant differences between MICT and control, *p* < 0.05; ^c^significant differences between HIIT and MICT, *p* < 0.05.

At baseline, there were no differences across groups in the body composition measurements (Figure 4). Following 6 weeks of exercise, the results showed that the HIIT group demonstrated significant improvements compared to MICT in waist circumference (Δ% HIIT: 4.1%, *p* = 0.002 vs. Δ% MICT: 2.5%, *p* = 0.002) and body fat mass (Δ% HIIT: 4.5%, *p* < 0.001 vs. Δ% MICT: 3.2%, *p* < 0.001). The control group had no improvements. On the other hand, all values of body composition measurements increased from baseline to postintervention. The respective ES from baseline to 6 weeks was small in the HIIT group in body weight (*d* = 0.20), abdominal fat percentage (*d* = 0.28), and BMI (*d* = 0.22) and medium in waist circumference (*d* = 0.34). Moreover, in the MICT group, the ES was small in body fat percentage (*d* = 0.22), total body fat mass (*d* = 0.22), and waist circumference (*d* = 0.22).

Following the 6 weeks supervised program, VO_2_peak significantly increased by 14% with HIIT (Δ = 2.5 ± 1.5 ml·kg^−1^·min^−1^, *p* < 0.001) and 9% with MICT (Δ = 1.4 ± 1.2 ml·kg^−1^·min^−1^, *p* < 0.001) (Figure 4). Moreover, the control group VO_2_peak decreased by 0.2% (Δ = −0.7 ± 1.3 ml·kg^−1^·min^−1^, *p* = 0.491). The respective ES from baseline to 6 weeks was large in HIIT (*d* = 1.54) and MICT (*d* = 0.68).

Regarding the maximal strength of the knee extensor and flexor variables (Figure 4), despite descriptive analysis demonstrating an increase of 13% at 6 weeks in the variable “isokinetic peak torque (extension 60°)” in HIIT (Δ = 11.9 ± 27.6 N·m, *p* = 0.007) and of 10% in MICT (Δ = 9.1 ± 22.8 N·m, *p* = 0.061), the control group had a decrease of 0.4% (Δ = −3.0 ± 22.8 N·m, *p* = 0.835, *d* = 0.07). The respective ES from baseline to 6 weeks was small in HIIT (*d* = 0.24) and MICT (*d* = 0.20). A positive increase between baseline and the 6 weeks was observed in the variable “isokinetic peak torque (flexion 60°)” in HIIT of 15% (Δ = 7.2 ± 14.2 N·m, *p* = 0.002) and MICT of 14% (Δ = 6.9 ± 16.0 N·m, *p* = 0.022). On the other hand, the control group decreased by mean 0.2% (Δ = −0.3 ± 12.8 N·m, *p* = 0.835). The respective ES from baseline to 6 weeks was small in HIIT (*d* = 0.29) and medium in MICT (*d* = 0.33).

**Figure 5:**
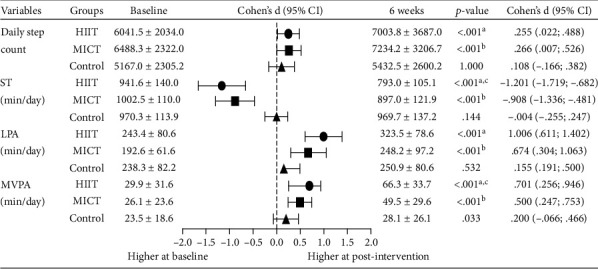
Physical activity and sedentary behavior levels of exercise groups (HIIT (*n* = 23) and MICT (*n* = 23)) and control group (*n* = 23). HIIT = high-intensity interval training (*n* = 23); MICT = moderate-intensity continuous training (*n* = 23); Control = control group (*n* = 23); ST = sedentary time (1.00–1.99 MET); LPA = light physical activity (2.00–3.49 MET); MVPA = moderate-to-vigorous physical activity ( ≥3.50 MET). Values are reported as mean ± standard deviation. ^a^Significant differences between HIIT and Control, *p* < 0.05; ^b^significant differences between MICT and Control, *p* < 0.05; ^c^significant differences between HIIT and MICT, *p* < 0.05.

Figure 5 presents the PA and SB of the exercise and control groups. Following the 6-week supervised program, HIIT decreased significantly the sedentary time (ST) of 15% (Δ = −148.6 ± 106.1 min/day, *p* < 0.001) and MICT decreased 10% (Δ = −105.5 ± 88.0 min/day, *p* < 0.001), and control decreased 0.1% (Δ = 0.559 ± 73.8 min/day, *p* = 0.144). Regarding the PA, HIIT increased the daily step count of 33% (Δ = 4162.3 ± 8339.7 step count, *p* < 0.001), MICT increased 10% (Δ = 745.9 ± 1605.4 step count, *p* < 0.001), and control increased 6.5% (Δ = 265.5 ± 1524.4 step count, *p* = 1.000). In LPA, HIIT increased 39% (Δ = 80.1 ± 45.2 min/day, *p* < 0.001), MICT increased 30% (Δ = 55.6 ± 60.3 min/day, *p* < 0.001), and control increased 9% (Δ = 12.6 ± 65.5 daily step count, *p* = 0.532). In MVPA, HIIT improved significantly 54% (Δ = 16.4 ± 14.4 min/day, *p* < 0.001), MICT improved 45% (Δ = 13.4 ± 12.4 min/day, *p* < 0.001), and control improved 19% (Δ = 4.5 ± 13.7 step count, *p* = 0.033). Significant differences were observed between the exercise and control groups in sedentary time, LPA, MVPA, and number of steps (*p* < 0.001). Between the exercise groups, HIIT showed significant improvements in sedentary behavior and moderate-to-vigorous PA compared to MICT (*p* < 0.001). The respective ES from baseline to 6 weeks in daily step count was small in HIIT (*d* = 0.26) and MICT (*d* = 0.27), ST was large in HIIT (*d* = 1.20) and MICT (*d* = 0.91), LPA was large in HIIT (*d* = 1.01) and MICT (*d* = 0.67), and finally, MVPA was small in control (*d* = 0.20) and large in HIIT (*d* = 0.70) and MICT (*d* = 0.50).

## 4. Discussion

To our knowledge, this study is the first randomized controlled trial to characterize the effects of 6-week community-based exercise protocols in CAD patients' health indicators such as physical fitness and physical activity levels. The main findings of our study are as follows: (i) physical fitness—HIIT and MICT exercise protocols promoted a significant improvement in VO_2_peak, body weight, BMI, body fat percentage, total body fat mass, abdominal fat percentage, and waist circumference, compared to the control group—and (ii) the physical activity improvement in patients undergoing HIIT protocol was more positive than MICT and mainly detected by diminution of sedentary time and increase of moderate-to-vigorous activity time. On the contrary, the control group decreased VO_2_peak, muscle strength, and PA and increased body composition variables and sedentary time from baseline to six weeks.

Our study demonstrated that HIIT and MICT significantly decreased most body composition variables compared with patients who did not undergo exercise-based community programs. It is well documented that exercise training disproportionately reduces visceral fat compared to total body fat stores [50], and exercise does appear superior to dieting for inducing visceral fat loss [51]. The tendency for the control group was an increase in abdominal fat (+1%), body fat mass (+0.5 kg), and waist circumference (+1.1 cm) after six weeks. These results require attention because body fat mass and abdominal fat percentage are associated with a higher risk of cardiovascular events and all-cause mortality [52, 53]. On the contrary, body composition was positively affected by the HIIT intervention. Patients in the HIIT group reduced their weight by a mean 1.9 kg (−3.1%) more than patients in the MICT group (mean −0.9 kg, −3%). Moreover, there was a moderate fat loss in both HIIT (mean −0.9 kg, −3%) and MICT (mean −0.9 kg, −3%) counteracted somewhat by a near-negligible increase in lean body mass in HIIT (mean +0.2 kg, 1.8%) and MICT (mean +0.2 kg, 0.5%). Furthermore, it is worth noting that both HIIT and MICT demonstrated a significant decrease in abdominal fat loss, with a mean reduction of 1.8% and 1.3%, respectively. This reduction is particularly important in reducing the risk of CVD. These results on body composition variables demonstrated a beneficial effect of a higher-intensity exercise session in an exercise based on body composition, which is in accordance with what has been shown by others [54–56]. For example, Dun et al. [57] compared HIIT and MICT and found that HIIT results in significant reductions in total fat mass and abdominal fat percentage in CAD patients compared to MICT. Trapp et al. [58] compared HIIT and MICT and discovered the same effects. They showed that the HIIT group had a greater decrease in abdominal fat. Still, Zhang et al. [59] demonstrated that both HIIT and MICT significantly reduced abdominal and total fat mass. However, our study duration of six weeks was a relatively short period of time. If the intervention had been longer, we would likely have observed a clinically relevant effect in this variable.

Aerobic capacity (VO_2_peak) improved by 14%, equivalent to 2.5 mL kg^−1^ min^−1^ or nearly 1 MET, in the HIIT group and 9% in the MICT group (1.4 mL kg^−1^ min^−1^) compared with the control group. The improvements of HIIT were almost twice as good as the MICT group (Δ = 2.3 ± 1.5 mL kg^−1^ min^−1^, *p* < 0.001, *d* = 1.54 vs. Δ = 1.4 ± 1.2 mL kg^−1^ min^−1^, *p* < 0.001, *d* = 0.68, respectively). Our results indicated that training intensity is essential in improving peak aerobic capacity in CAD patients. Moreover, the mean between HIIT and MICT of 0.9 mL kg^−1^ min^−1^ could be considered clinically meaningful as each 1 mL kg^−1^ min^−1^ improvement in VO_2_peak during a CR program has been associated with an ∼8–17% reduction in all-cause and cardiovascular-related mortality [16, 30–33]. Furthermore, Du et al. [60] concluded that studies that used a nonisocaloric exercise protocol induced greater gains in VO_2_peak compared to studies that used an isocaloric exercise protocol, indicating that the benefits of aerobic capacity can be determined by total caloric consumption. This is explained by the fact that we did not have greater results in this variable since we projected the same caloric expenditure between the two training intensities. Our study is consistent with findings from Keteyian et al. [61], a study including 2812 cardiac patients, which showed that an increase of 1 mL kg^−1^ min^−1^ in VO_2_peak can reduce the cardiovascular-specific mortality risk by 15%. Moreover, the greater efficacy of HIIT for improving VO_2_peak compared with MICT during supervised training is similar to previous meta-analyses reporting group differences of 1.5 to 1.6 mL kg^−1^ min^−1^ [62–65]. Similarly, Rognmo et al. [66] demonstrated that HIIT was effective in improving aerobic capacity in CAD patients. In addition, in our previous meta-analysis of 16 studies (*n* = 969 patients), we found that moderate-to-vigorous (SMD = 1.84 mL kg^−1^ min^−1^; 95% CI [1.18, 2.50]) and vigorous-intensity (SMD = 1.80 mL kg^−1^ min^−1^; 95% CI [0.82, 2.78]) exercise interventions resulted in larger increases in relative VO_2_peak compared to moderate-intensity exercise interventions (SMD = 0.71 mL kg^−1^ min^−1^; 95% CI [0.27, 1.15]) [20]. Sandercock et al. [67] observed greater improvements of 5.2 mL kg^−1^ min^−1^ (95% CI: 4.1–6.4) in CAD patients, and Uddin et al. [68] presented improvements of 3.3 mL kg^−1^ min^−1^ (95% CI: 2.6–4.0).

However, the control group who did not undergo community-based exercise programs decreased VO_2_peak by −0.2% (Δ = −0.7 ± 1.3 mL kg^−1^ min^−1^, *p* = 0.491, *d* = 0.07) which is alarming since it has been recognized that aerobic capacity can be a strong predictor of both cardiovascular and all-cause mortality [69]. Fortunately, a 12-week exercise-based CR program can significantly improve aerobic capacity, as shown by Martin et al. [70]. This same study also indicated that for each metabolic equivalent increase in VO_2_peak, there was an overall reduction in mortality of 13%. Moreover, patients who began the program with a low fitness level experienced a 30% decrease in mortality [70].

Impaired muscle strength is powerfully related to poor exercise capacity [71, 72] and mobility disability [73] and predicts a higher rate of mortality [39] in CAD patients. At baseline, we found that the muscle strength of all groups was low. These results are consistent with previous studies in CAD patients before exercise-based programs [9, 74]. After six weeks, our study demonstrated that HIIT and MICT increased muscle strength compared with patients who did not undergo community-based exercise programs. However, HIIT increased muscle strength more than the MICT group. However, no significant increase was observed in our study, which is expectable because we only focused on aerobic training and we did not prescribe exercises for resistance training. The training effect on muscle strength in our study was similar to that demonstrated by Kida et al. [75]. Yamamoto et al. [76] reported an increased muscle volume in CAD patients, but no significant increase was observed in the study too.

In general, physical fitness (body composition, aerobic capacity, and muscle strength) in both community-based exercise programs in 6 weeks improved, which was similar to other studies [75, 77–80]. For example, Beniamini et al. [77] demonstrated that HIIT during the 12-week CR program improved aerobic capacity and muscle strength and changed body composition. However, Fragnoli-Munn et al. [79] reported an improvement in aerobic capacity and muscle strength but not in body composition. Pierson et al. [80] reported mean percent strength increase 44 to 81% and significantly increased in the VO_2_peak within both groups after training, but the relative improvement between groups was not different. Our results show that the control group displayed a lack of changes or even degradation of physical fitness (e.g., VO_2_peak), suggesting the critical importance of referring CAD patients to a community-based exercise program.

Physical activity is a crucial component of CR programs, which focus on reducing SB and increasing MVPA [81]. Nevertheless, only a few studies have objectively measured PA and SB before enrollment in CR [82–87]. Our results demonstrate high levels of SB in all three groups prior to enrollment, and their daily routine consists mainly of LPA. That is alarming since SB is an important and independent risk factor for CVD. Moreover, these results are consistent with previous findings when entering community-based exercise programs. Patients with CAD were found to be mostly sedentary, spending around 10.5 to 12 hours per day in a sitting position. They also spent 3.5 hours per day in LPA and rarely engaged in MVPA before enrolling in CR (20 to 65 minutes per day) [81–83, 85, 86]. The recent World Health Organization PA guidelines recommend that adults accumulate as much MVPA as possible throughout the day, regardless of the single bout duration [88]. After six weeks, we found a significantly higher level of daily MVPA (+36 min/day, *p* < 0.001) in HIIT and MICT (+23 min/day, *p* < 0.001) compared with the control group. However, we obtained similar results when comparing HIIT and MICT in relative daily LPA. Our findings are similar to previous studies with a CAD population [89–91]. Previously, both LPA and MVPA were related to lower CVD risk [92]. In our study, we found that HIIT spent more time in MVPA and less time being sedentary compared to MICT. According to PA guidelines, adults should spend at least 150 minutes per week engaging in MVPA [88, 93]. Adhering to these guidelines has been linked to a lower risk of all-cause and cardiovascular mortality risk despite a previously inactive lifestyle [94]. For MVPA and ST, this is partially in line with our results, as patients in our study performed slightly more MVPA and were less sedentary compared to some prior research [93, 95]. Besides, a greater amount of daily PA at any intensity level and avoiding ST are recommended.

In our study, we show a positive association between MVPA and aerobic capacity in patients after enrollment in community-based exercise programs. For the betterment public health, it is crucial that individuals with high sedentary behavior should at least increase their LPA to enhance their cardiovascular health and lower the risk of mortality. As aerobic capacity is a strong predictor of mortality in CAD patients [96], future epidemiological or interventional studies should accurately evaluate the impact of PA and sedentary behavior on clinical outcomes like mortality and rehospitalization. Additionally, female patients should be included in these studies to provide further evidence on their physical fitness and levels of PA.

To finalize, our results suggest that HIIT has a clinically significant effect in improving physical fitness and physical activity in CAD patients without adversely affecting patient safety. There were no adverse events in either protocol (HIIT and MICT) during the exercise interventions. Only one patient from each group discontinued the intervention, achieving 96% adherence in both groups, HIIT and MICT protocols. The observed positive efficacy findings are highly encouraging, particularly considering the significant changes that were induced within a relatively short duration of just 6 weeks and with a low training frequency of only 3 sessions per week, totaling approximately 18 sessions per patient. Chaves et al. [36] suggest that the ideal duration of community-based exercise intervention is between 12 and 36 sessions. Hence, our study demonstrated that HIIT was considered a beneficial and feasible supplementary therapy in community-based exercise program to MICT like other multiple large-scale epidemiological studies reporting the same [97].

This study includes certain limitations that should be acknowledged. Firstly, the relatively small sample size raises the possibility that only more substantial differences would attain statistical significance. Secondly, the unintended gender bias observed in the patient cohort, with only 13–17% representation of women, poses a limitation in terms of the generalizability of the findings. It is important to note that the sex distribution in the study was an unintended consequence of our clinical population composition. When considering the results of this study, due consideration must be given to potential confounding effects stemming from concurrent medications, although it is crucial to highlight that no alterations in medication dosages for lipid-lowering and heart rate control occurred throughout the study duration. Furthermore, it is noteworthy that the control group participants were not provided with diaries, thereby rendering us devoid of information regarding their physical activity patterns during the intervention period spanning from baseline to the six-week mark. The potential increase in physical activity within the control group could introduce a mitigating factor, potentially diminishing the observed differences in effects between the various groups.

## 5. Conclusions

In conclusion, this RCT showed that both 6-week HIIT and MICT programs were safe and effective to promote beneficial effects on the patient's physical fitness (body composition, aerobic capacity, and muscle strength) and physical activity. More importantly, compared to conventional exercise-based programs (MICT), the HIIT group showed further improvements in VO_2_peak for reducing total body fat mass, abdominal fat percentage, waist circumference, and sedentary behavior and improving the MVPA in CAD patients. However, not doing any type of exercise-based following a cardiac event has shown worse results in all studied clinical variables. Importantly, no adverse event was detected, so these findings support HIIT as a beneficial adjunct or alternative to MICT in community-based exercise programs and should be considered an important treatment strategy for CAD patients.

## Figures and Tables

**Table 1:** Baseline characteristics of the study participants.

	Exercise-based program		Nonexercise-based program
	HIIT (*n* = 23)	MICT (*n* = 23)	Control (*n* = 23)
Demographics			
Age (years), mean ± SD	50 ± 9	55 ± 10	57 ± 11
>70 years, **n** (%)	2 (8.7)	3 (13.0)	4 (17.4)
Gender (male/female)	20/3	19/4	20/3
Retired, **n** (%)	2 (8.7)	7 (30.4)	7 (30.4)
Anterior MI, **n** (%)	3 (13.0)	4 (17.4)	2 (8.7)
Coronary event/intervention			
CABG, **n** (%)	1 (4.3)	1 (4.3)	1 (4.3)
PCI, **n** (%)	22 (95.7)	22 (95.7)	22 (95.7)
Risk factors or comorbidities			
Diabetes mellitus, **n** (%)	10 (43.5)	9 (39.1)	10 (43.5)
Hypertension, **n** (%)	13 (56.5)	13 (56.5)	14 (60.9)
Dyslipidemia, **n** (%)	14 (60.9)	15 (65.2)	15 (65.2)
Body mass index (kg/m^2^), mean ± SD	28.2 ± 4.5	29.4 ± 3.9	29.4 ± 4.3
Waist circumference (cm), mean ± SD	98.4 ± 14.5	101.1 ± 10.3	101.1 ± 10.8
Active smoker, **n** (%)	6 (26.1)	4 (17.4)	4 (17.4)
Nonsmoker, but has been, **n** (%)	9 (39.1)	13 (56.5)	12 (52.2)
Family history of CVD, **n** (%)	14 (60.9)	16 (69.6)	16 (69.6)
Sedentarism, **n** (%)	13 (56.5)	19 (82.6)	19 (82.6)
Sleep < 5 h, **n** (%)	6 (26.1)	9 (39.1)	11 (47.8)
Current medication			
ACE inhibitor, **n** (%)	21 (91.3)	23 (100)	22 (95.7)
ARBs, **n** (%)	16 (69.6)	7 (73.9)	11 (47.8)
Antiplatelet, **n** (%)	22 (95.7)	22 (95.7)	23 (100)
CCBs, **n** (%)	2 (8.7)	5 (21.7)	5 (21.7)
Beta-blockers, **n** (%)	21 (91.3)	22 (95.7)	22 (95.7)
Diuretics, **n** (%)	2 (8.7)	4 (17.4)	6 (26.1)
Insulin, **n** (%)	5 (21.7)	5 (21.7)	11 (47.8)
Statin, **n** (%)	22 (95.7)	22 (95.7)	23 (100)

ACE = angiotensin-converting enzyme inhibitor; ARBs = angiotensin II receptor blockers; CCBs = calcium channel blockers; HIIT = high-intensity interval training; MI = myocardial infarction; MICT = moderate-intensity continuous training; VO_2_peak = maximal oxygen consumed. Data are reported as mean ± standard deviation or number and percent population (%). Significance is <0.05.

## Data Availability

The data that support the findings of this study are available from the corresponding author, C.G., upon reasonable request.
